# Polyphenol-Rich Ginger (*Zingiber officinale*) for Iron Deficiency Anaemia and Other Clinical Entities Associated with Altered Iron Metabolism

**DOI:** 10.3390/molecules27196417

**Published:** 2022-09-28

**Authors:** Soo Liang Ooi, Sok Cheon Pak, Ron Campbell, Arumugam Manoharan

**Affiliations:** 1 School of Dentistry and Medical Sciences, Charles Sturt University, Bathurst, NSW 2795, Australia; 2 The Oaks Medical Practice, The Oaks, NSW 2570, Australia; 3 Graduate School of Medicine, University of Wollongong, Wollongong, NSW 2522, Australia

**Keywords:** blood disorder, haemoglobin, natural product, nutraceutical, nutritional disease, phenolic compounds

## Abstract

Ginger (*Zingiber officinale*) is rich in natural polyphenols and may potentially complement oral iron therapy in treating and preventing iron deficiency anaemia (IDA). This narrative review explores the benefits of ginger for IDA and other clinical entities associated with altered iron metabolism. Through in vivo, in vitro, and limited human studies, ginger supplementation was shown to enhance iron absorption and thus increase oral iron therapy’s efficacy. It also reduces oxidative stress and inflammation and thus protects against excess free iron. Ginger’s bioactive polyphenols are prebiotics to the gut microbiota, promoting gut health and reducing the unwanted side effects of iron tablets. Moreover, ginger polyphenols can enhance the effectiveness of erythropoiesis. In the case of iron overload due to comorbidities from chronic inflammatory disorders, ginger can potentially reverse the adverse impacts and restore iron balance. Ginger can also be used to synthesise nanoparticles sustainably to develop newer and more effective oral iron products and functional ingredients for IDA treatment and prevention. Further research is still needed to explore the applications of ginger polyphenols in iron balance and anaemic conditions. Specifically, long-term, well-designed, controlled trials are required to validate the effectiveness of ginger as an adjuvant treatment for IDA.

## 1. Introduction

Anaemia develops when the body’s circulating erythrocytes or red blood cells (RBCs) fall below normal. According to the guidelines of the World Health Organization (WHO), anaemia is diagnosed with haemoglobin (Hb) concentration lower than the current cut-off level, defined as Hb <130 g/L for adult males, <120 g/L for non-pregnant women, and <110 g/L for children (6–59 months) [1]. The reduction in RBCs may lead to insufficient oxygen-carrying capacity of the blood to meet physiological needs, resulting in symptoms such as fatigue, weakness, shortness of breath, chest pain, reduced physical tolerance and restless leg syndrome [2]. In adults, anaemia may lead to increased morbidity and decreased work productivity and poor birth outcomes during pregnancy. In children, anaemia can cause impaired cognitive and behavioural development, and even increase mortality [1].

In 2019, the worldwide prevalence of anaemia for all ages was 22.8% (95% confidence interval (CI): 22.6–23.1) [3]. The global burden of disease measured in years living in disability for anaemia is 672.4 (95% CI: 447.2–981.5) per 100,000 population [4]. Women and children in low-income countries are the most vulnerable groups. This condition affected 29.9% (95% CI: 27.0–32.8) of women of reproductive age, of which 36.5% (95% CI: 34–39.1) of pregnant women suffered from anaemia compared to 29.6% (95% CI: 26.6–32.5) of non-pregnant women [5]. About 269 million children under five also had anaemia, with a global prevalence of 39.8% (95% CI: 36.0–43.8). The highest was in the African region, affecting 60.2% (95% CI: 56.6–63.7) of children [5].

Even in a wealthy society like Australia, anaemia remains a health risk. According to the Australian Health Survey last conducted in 2011–2012, 4.5% of the population aged 18 years and over had anaemia, with women having a relative risk of 2.56 times more than men [6]. The burden of anaemia is even higher among the Australian indigenous population, with a female-specific prevalence of 15.3% [7], signifying health inequality due to socioeconomic consequences.

Anaemia is not a disease per se but a manifestation of other underlying causes. Iron deficiency, which results from increased iron demands, deminished iron supply, blood loss or malabsorption of iron, is the most common cause of anaemia worldwide, accounting for nearly two-thirds of global anaemia cases [4,8]. Hence, iron deficiency anaemia (IDA) is a global health concern affecting millions worldwide, especially women and children in less developed regions. IDA is routinely treated with oral iron supplements such as ferrous sulphate but compliance issues due to gastrointestinal side effects often hamper its effectiveness [9,10].

In the broader context, ginger plants refer to all perennial flowering plants in the Zingiberaceae family, which include many aromatic herbs and spices such as turmeric, cardamom, and galangal. Currently, there are 1888 unique species in the Zingiberaceae family classified into 62 genera, of which 204 species belong to the *Zingiber* genera [11]. The ginger root commonly consumed worldwide is the rhizome of *Zingiber officinale* species. Accordingly, in this review, ginger only refers to the *Z. officinale* species.

*Z. officinale* has a long history of culinary and medicinal use, possibly even before formally recorded history. Wu [12] suggested that ginger cultivation originated around the Yangtze River and Yellow River basins in ancient China. However, this claim has yet to be widely accepted. The spice trade spread ginger to major civilisations from East Asia and India to the Greek, the Roman Empire, and beyond. Today, ginger is primarily used as a food, spice, herb, and flavouring agent, with a global trade volume of USD 1.06 billion in 2019, with China being the top exporter supplying over 57.8% of the world demand [13]. Three varieties of ginger are consumed as food and herbs: white (var. Roscoe), small white (var. Amarum) and red (var. Rubra), with *Z. officinale* Roscoe being the most common variety [14].

Ginger possesses several health-promoting properties and has been traditionally used in East Asia to ease fatigue and weaknesses. Contemporarily, ginger is considered a functional food that can confer health benefits beyond its nutritional values for preventing, managing, or treating disease [15,16,17,18]. As a rich source of natural polyphenols, ginger may potentially complement oral iron therapy in treating IDA and be a supportive dietary strategy for preventing IDA. Hence, there has been heightened commercial interest in using ginger, especially in China, as an ingredient for functional foods or ethnomedicine for IDA, as evidenced by the growing number of patents filled with the World Intellectual Property Organization in recent years (see Table A1 in Appendix A). However, there is a lack of research literature critically reviewing such potentials.

The objective of this narrative review is to inform translational research on the benefits of ginger and its bioactive polyphenols in the context of IDA and other clinical entities associated with altered iron metabolism based on available research. The ensuing sections first examine ginger’s application as a functional food, followed by the pathophysiology of IDA and its treatment. These overviews provide context to support the subsequent review of the various beneficial properties of ginger and its polyphenols applicable to IDA based on pre-clinical and clinical evidence. To the authors’ knowledge, this is the first attempt to comprehensively investigate the scope and depth of current literature on this underexplored topic.

## 2. Ginger as a Functional Food

### 2.1. Nutritional Composition and Traditional Use

Nutritional analysis has shown that ginger consists mainly of moisture, carbohydrate, protein, fibre, fat, and ash. It is rich in polyphenols and contains micronutrients including ascorbic acid, β-carotene, calcium, iron, and copper. However, it is worth noting that the nutritional composition of ginger can vary greatly depending on the varieties, origin, time of harvest, drying method, and storage condition. Table 1 shows the approximate nutritional composition of dried ginger powder reported in the literature [19,20]. Ginger is, however, valued beyond its nutritional benefits. It is believed that the Indian and Chinese populations have used ginger as a tonic for over 5000 years [21]. In Shen Nong Ben Cao Jing, the oldest surviving Chinese materia medica circa 100BC, ginger was classified as a middle category of herb with little or no toxicity and was mainly used in combination prescription to treat deficiency to prevent illness or resist worsening disease [22]. Incidentally, ginger is also used in traditional Ayurvedic medicine to treat many diseases such as diabetes, flatulence, intestinal colic, indigestion, infertility, inflammation, insomnia, nausea, rheumatism, stomach ache, and urinary tract infections [23].

### 2.2. Phytochemistry and Health Benefits

With increasing interest in the therapeutic applications of natural products, ginger has received much attention in recent years, with numerous studies and reviews exploring its phytochemistry, pharmacological, and health-benefiting properties [14,21,22,23,24,25,26]. Overall, ginger is rich in phytochemicals, with over 300 identified constituents divided into three main categories: gingerols, volatile oils, and diarylheptanoids, as reported by Liu et al. [27]. The most notable are the phenolic compounds in gingerols, shogaols, and paradols, including 6-gingerol, 6-shogaol, 8-gingerol, 8-paradol, 10-gingerol, and many more [28]. These compounds are responsible for the unique pungent smell and taste of ginger. Other gingerol-related compounds include zingerone, gingerenone A, and 1-dehydro-10-gingerdione [29]. In addition, the volatile oils of ginger also contain terpene compounds such as β-bisabolene, α-curcumene, zingiberene, α-farnesene, and β-sesquiphellandrene [30]. The ginger’s diarylheptanoid contents can be divided into linear diphenyl heptane and cyclic diphenyl heptane compounds with antioxidant activity [27].

**Figure 1:**
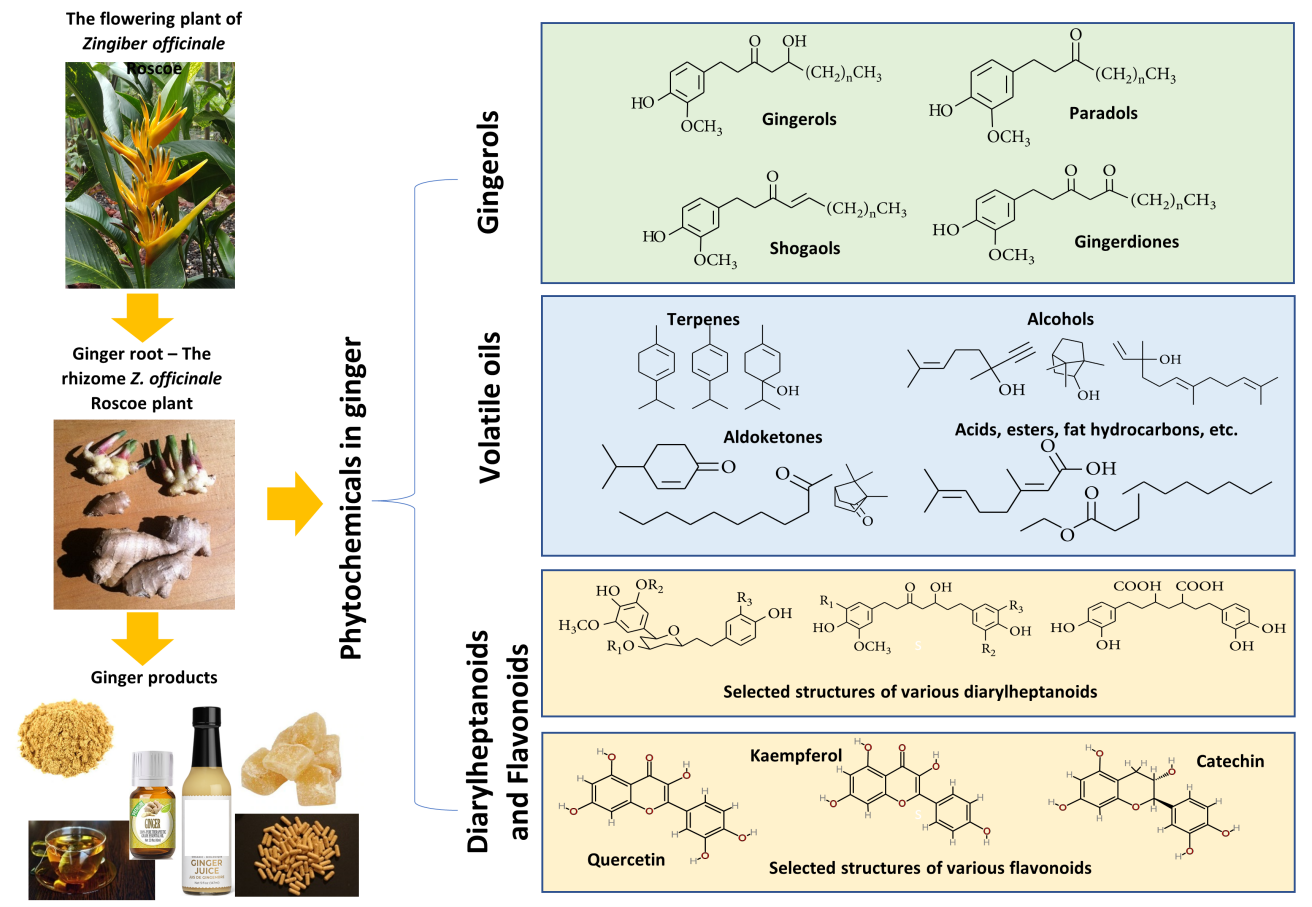
An overview of ginger and its main groups of active constituents of gingerols, volatile oils, diarylheptanoids, and flavanoids with some sample structural formulas. In the formulas of gingerols, _n_ denotes the number of repeating units of CH_2_. R_1_, R_2_, and R_3_ in the selected diarylheptanoid structures are sites for functional groups or substituents (e.g., H, CH_3_, OCH_3_, or COCH_3_).

Ginger is also known to contain many secondary metabolites of flavonoids and other phenolic components such as quercetin, rutin, catechin, epicatechin, kaempferol, naringenin, fisetin, morin, hesperidin, salicylic acid, and chlorogenic acid [31,32,33]. The concentrations of these secondary metabolites in ginger may vary significantly across samples as their contents are influenced by the environmental conditions (including light intensity, temperature, insects, etc.) where ginger is grown and methods of drying and storage. Higher phenolic and flavonoid content in ginger is known to increase its antioxidant activities [34,35]. Figure 1 provides an overview of ginger and its constituents.

Among these constituents, 6-gingerol is the most pharmacologically active compound [26], whereas 6-shogaol has a higher potency than 6-gingerol in terms of bioactivities but exists in a lesser quantity naturally [36]. By studying ginger’s typical metabolic pathways in a mouse model, the in vivo effects were found to derive mainly from 6-gingerol and 6-shogaol, with hydrogenation, demethylation, glucuronidation, sulfation, and thiolation being their major metabolic reactions [16]. Shogaols are metabolised through more complicated pathways than gingerols, and these two compounds have different molecular targets even though both are reported to have potent antioxidant and anti-inflammatory actions [37].

The pharmacological effects of ginger and its active compounds have been recently reviewed by many authors, including Unuofin et al. [23], Mao et al. [28], and Choi et al. [38]. Notable effects include anti-diabetic [39,40,41], anti-emetic [42,43,44], anti-nauseant [45,46], anti-obesity [47,48,49,50,51], anti-inflammatory [52,53,54,55], antioxidant [56,57,58,59,60], nephroprotective [61,62,63], neuroprotective [64,65,66], gastroprotective [67,68], and anti-melanogenesis [69,70] effects. Moreover, ginger may also be protective against male infertility [71]. The anti-inflammatory and antioxidant activities of ginger extract, and 6-gingerol in particular, have also been shown to have antiproliferative and anticancer properties in another review by de Lima et al. [26]. For clinical application, a systematic review of 109 randomised controlled trials (RCTs) by Anh et al. [25] found evidence to support the use of ginger for improvement in nausea and vomiting in pregnancy, inflammation, metabolic syndromes, digestive function, and colorectal cancer markers.

### 2.3. The Growing Popularity of Ginger

Ginger is widely used for food processing in many forms, including as fresh ginger and dried ginger, as an oleoresin, as an essential oil, as an extract, or as a powder. The addition of ginger to food gives a spicy taste and serves as a natural antioxidant for shelf-life extension [72,73]. Furthermore, with many health-promoting properties supported by research evidence, it is not surprising that ginger is also a choice ingredient in various kinds of functional foods, such as candy, biscuits, herbal tea, and beverages, etc. Most notably, ginger candy is used to reduce the vomiting frequency among pregnant women in the first trimester [74]. Ginger tea, a spicy caffeine-free alternative to black tea or coffee, has also emerged as one of the most popular beverages in the world [75]. Ginger supplements also ranked 6th in the American top-selling herbal chart of the mainstream retail channels in 2020, with over USD 64 million in sales, a 39.3% growth compared to sales volume in 2019 [76]. Undeniably, the popularity of ginger as a functional food will continue to spread with the growing consumer awareness of its health benefits.

### 2.4. Safety

Safety data from an animal study showed that consuming up to 1 g/kg body weight per day of a standardised ethanol extract of ginger had no significant effects on blood glucose, blood coagulation, blood pressure, and heart rate in rats compared to controls [77]. Long-term (35 days) force-feeding of rats with ginger powder up to 2 g/kg body weight was also not associated with any mortality or abnormalities in general conditions, behaviour, growth, and food and water consumption, as shown by Rong et al. [78]. Acute and subacute toxicity studies in rats with an enriched ginger extract (8% gingerols) reported no mortality or clinical signs of toxicity at a dose level of 2000 mg/kg (LD_50_ > 2000 mg/kg). The repeated administration of ginger extract for 28 days in rats at 1000 mg/kg also did not induce any observable toxic effects, with the no observed adverse effect level (NOAEL) calculated as 1000 mg/kg daily [79].

An oral toxicity study of ginger essential oil (31% zingiberene) in Wistar rats found no adverse effect after 13 weeks of subchronic oral administration. The NOAEL for ginger essential oil was determined to be over 500 mg/kg per day [80]. In another study, Idang et al. [81] performed toxicological assessments of both ginger essential oil and ginger fixed oil in Wistar rats for 60 days and found signs of increased oxidative stress and some forms of pathologies in the livers and spleens of the experimental animals fed up to 0.2 mL/kg of ginger fixed oil. Hence, the authors cautioned against the long-term use of fixed oils derived from ginger. Additional study to validate and confirm the toxicity of ginger fixed oil is required.

In pregnant rats, Weidner and Sigwart [82] also showed that feeding with 1 g/kg body weight of a standardised ginger extract did not cause any maternal or developmental toxicity. In contrast, Wilkinson [83] reported that pregnant Sprague-Dawley rats’ exposure to ginger tea increased early embryo loss but enhanced growth in surviving foetuses. Notwithstanding, a systematic review of 14 RCTs and 3 prospective clinical studies over more than 25 years found that ginger use during pregnancy does not pose a risk for the mother and the foetus [84]. Therefore, ginger consumption has no safety concern during pregnancy.

### 2.5. Adverse Events

In humans, a daily intake of ginger up to four grams is generally considered safe [85]. However, consumption at doses higher than six grams may increase the risk of gastrointestinal disturbances such as gastrointestinal reflux, heartburn, and diarrhoea [86]. In a systematic review of RCTs by Anh [25], 17 of 43 high-quality included trials provided adverse event information. Heartburn was the most common adverse event reported in 16 studies. Other gastrointestinal disturbances reported with ginger treatment were abdominal pain, bloating, gas, and epigastric distress. None of the events were considered severe [25].

Rare cases of allergic rhinoconjunctivitis were described in the literature concerning occupational exposure to ginger. For instance, Schmidt et al. [87] reported that a 60-year-old woman developed allergic rhinoconjunctivitis with red eyes, sneezing, nasal congestion and a runny nose due to exposure to dust particles containing ginger in her workplace. Similar incidences were reported in other occupational settings as well, with positive skin-prick tests against ginger confirming the reactions to be an immunoglobulin E antibody-mediated allergy [88,89]. The cysteine proteinase GP-I in ginger is thought to be the relevant allergen in these cases [90]. Moreover, occupational allergic contact dermatitis caused by ginger and other spices has also been reported [91]. Hence, ginger could contribute to allergic reactions, especially in patients with known hypersensitivity to spices.

Other potential side effects of ginger, when taken in large doses, may include prolonged pre-existing bleeding, central nervous system depression, and arrhythmia [86]. Therefore, properly dosing concentrated forms of ginger extract and derivatives are essential.

### 2.6. Drug Interactions

Ginger may also potentially interfere with the bioavailability of many oral pharmaceutical substances, either by increasing their absorption from the gastrointestinal tract or preventing their metabolism in the liver after absorption [92,93].

The most notable is the potential interaction between ginger and anticoagulant therapy as ginger is known to possess anti-platelet aggregation properties. Some case reports have suggested that ginger may interact with warfarin and increase the risk of prolonged bleeding [94,95,96,97]. Additionally, a prospective longitudinal study reported that ginger was associated with an increased risk of self-reported bleeding among patients taking warfarin, even though no significant risk of increasing blood clotting time was found [98]. However, a study investigating the effect of ginger on the pharmacokinetics and pharmacodynamics of warfarin in healthy volunteers co-administered with 0.4 g of ginger extract did not find evidence of interactions [99]. Furthermore, the effect of ginger on platelet aggregation and coagulation remains equivocal, according to a systematic review by Marx et al. [100] that included eight clinical trials and two observational studies. Hence, the European medicines agency did not find the case reports on the potential interactions between ginger and warfarin to be convincing [101].

Studies have shown that ginger may potentially interact with drugs such as crizotinib (an anti-cancer drug) [102], cyclosporine (an immunosuppressant) [103], metronidazole (an antibiotic) [104], and ketoconazole (an antifungal) [105]. With the increased use of ginger and its derivatives as nutraceuticals, more research is needed to identify and confirm potential ginger-drug interactions to reduce and avoid side effects induced by unfavourable interactions.

## 3. Pathophysiology of IDA and Its Treatment

### 3.1. Iron Homeostasis

**Figure 2:**
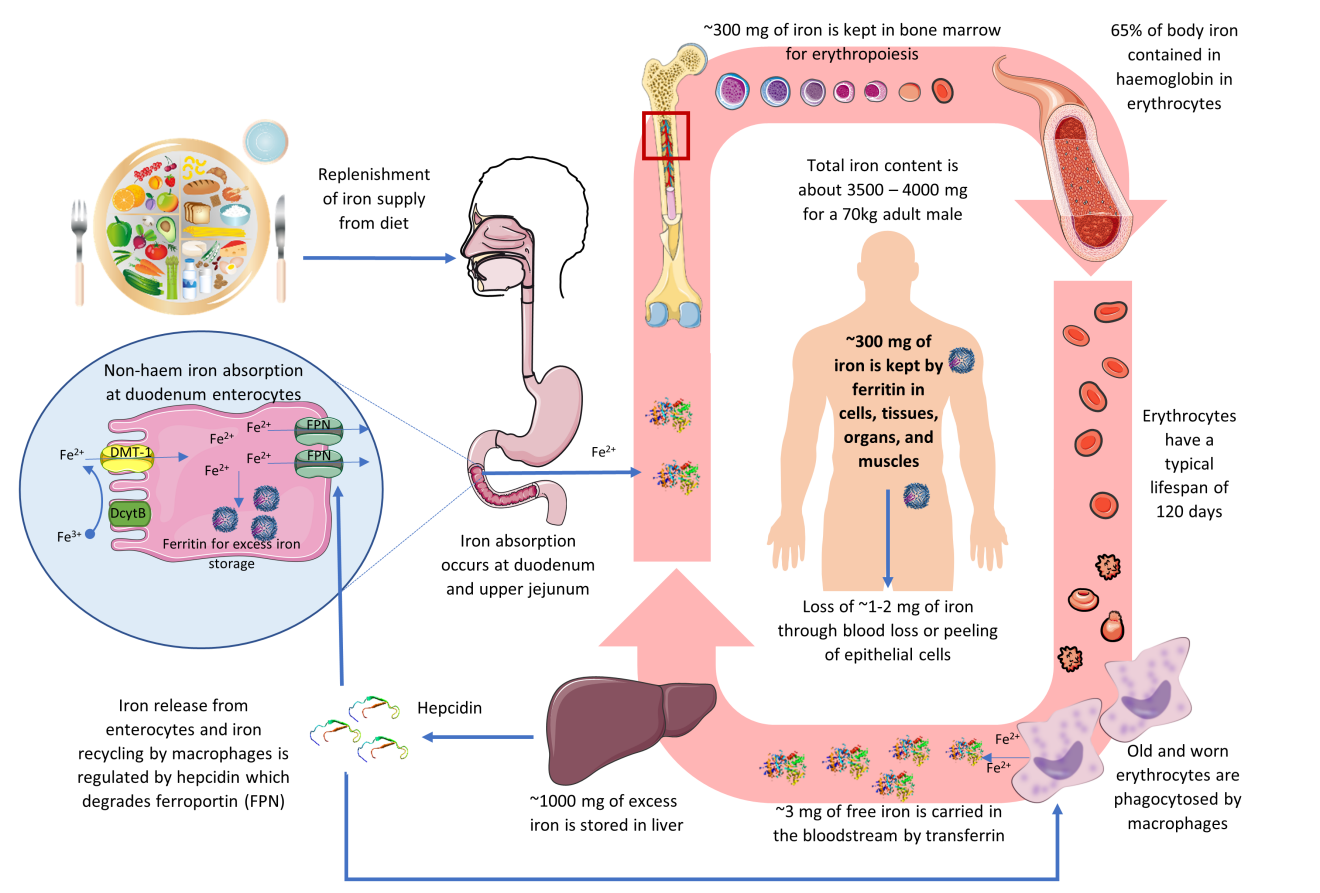
An overview of the iron cycle in humans depicting iron absorption, transportation, functioning, recycling, storage, and regulation.

The pathophysiology of IDA is closely related to the iron balance in the body. Iron is a vital trace mineral that plays a crucial role in many body functions, including oxygen transport, immune regulation, and enzyme catalyse reactions [106]. An overview of the iron cycle is depicted in Figure 2.

The total iron content in the body for a 70 kg adult male is about 3500 mg to 4000 mg, corresponding to a per kg bodyweight content of approximately 50 mg to 60 mg [106]. Around 65% of iron is contained within the Hb of RBCs to carry oxygen around the body [107]. RBCs have a typical life span of about 120 days. Old and worn RBCs are phagocytosed by the reticuloendothelial system, particularly the macrophages, in the spleen, liver, and bone marrow to release iron. In ferrous ion (Fe^2+^) form, about 3 mg of free iron is carried in the bloodstream by transferrin to the bone marrow to be recycled into making new RBCs. Approximately 300 mg of iron is kept in bone marrow for erythropoiesis [108,109]. Excess iron of up to 1000 mg is stored in the liver hepatocytes in complex hemosiderin. As ferritin, about 300 mg of iron is also contained in cells and tissues of organs and muscles. Together, transferrin, ferritin, and hemosiderin store approximately 20 to 30% of the iron in the body as spare [108,109].

The body loses about 1 to 2 mg of iron each day through blood loss or peeling of epithelial cells. Such loss is unregulated as humans and mammals have no iron excretion mechanism and must be replenished via controlled uptake from diet to maintain iron balance [109]. Diet is the only source of iron supply for the body after birth, excluding exogenous therapeutic means. There are two types of dietary iron, animal-derived haem iron and non-haem iron, the dominant form of iron in plants and abundant in both animal-based and plant-based foods [110]. The organic haem iron, which accounts for about 10% of the dietary iron, is more readily absorbable by the body [108], but its uptake mechanisms remain unclear, with receptor-mediated endocytosis and direct haem transporters of intestinal enterocytes being the two most prevailing hypotheses [108,110,111,112].

The inorganic non-haem iron, which makes up 90% of the total iron in food, is absorbed in the duodenum and upper jejunum section of the small intestines through a complex process [108]. Non-haem iron can be in Fe^2+^ or ferric (Fe^3+^) forms. First, Fe^3+^ must be reduced to Fe^2+^ ion by duodenal cytochrome B (DcytB), a trans-plasma membrane ferric reductase enzyme. Fe^2+^ can then traverse into the cytoplasm of the duodenal enterocytes by the divalent metal transporter 1 (DMT-1), the hydrogen-driven metal transporter residing at the apical membrane. An acidic micro-environment and the presence of ascorbate can facilitate the reduction of Fe^3+^ to Fe^2+^ and iron uptake across the apical membrane [106]. Iron in the enterocytes can be sequestered within the iron-buffering protein ferritin for later use. When iron is required by the body, it can be exported across the enterocyte basolateral membrane via ferroportin (FPN), an iron-regulated transporter protein. Iron stored as ferritin will be lost if not released by FPN as the enterocyte is sloughed after several days [110].

The body maintains its iron content in a tight balance; even though iron is physiologically essential, too much iron is toxic and detrimental to health. Systemic iron homeostasis is controlled through hepcidin, a liver peptide serving as the principal regulator [112]. Hepcidin induces FPN degradation to prevent export from iron-absorptive enterocytes and iron-recycling macrophages. High iron loading and inflammation will transactivate the hepcidin antimicrobial peptide (HAMP) gene in hepatocytes to encode hepcidin to reduce iron entry into circulation. In contrast, low iron status and increased erythropoietic demand will inhibit hepcidin transcription and promote iron export via FPN [109,112].

### 3.2. IDA and Its Aetiology

Iron deficiency anaemia can result from insufficient intake from the diet, decreased absorption, or blood loss [113]. Age, sex, lifestyle, and socioeconomic status may influence the adequacy of dietary iron intake. For example, growth spurts in children and adolescents or pregnancy may increase iron demand and cause iron deficiency without increased consumption of iron-rich foods. Poverty and poor diet can lead to malnutrition with low iron intake [114]. Decreased absorption can be due to dietary factors (e.g., high phytate diet or improper vegetarian or vegan diet), surgery, or gastrointestinal conditions (e.g., coeliac disease, inflammatory bowel disease, or gastritis) [113]. Blood loss due to heavy menstrual bleeding commonly leads to IDA in premenopausal women. Other sources of blood loss, such as injury, surgery, or occult gastrointestinal tract bleeding, also deplete the available RBCs and cause IDA [115].

There is also a reciprocal relationship between iron deficiency and inflammation, as shown in a 3-year prospective longitudinal study of 2141 relatively healthy older adults aged 70+ [116]. Posthoc analysis of the high sensitivity C-reactive proteins (CRP) and interleukin (IL)-6 levels, measured at 12, 24, and 36 months of follow-up, found baseline iron deficiency was associated with a more significant increase in IL-6 levels (mean difference in change: 0.52 ng/L, 95% CI: 0.03–1.00, *p* = 0.04) over 3 years. Additionally, iron deficiency at any yearly time point was associated with higher increases in CRP (mean difference in change: 1.62 mg/L, 95% CI: 0.98–2.26, *p* < 0.001) and IL-6 levels (mean difference in change: 1.33 ng/L, 95% CI: 0.87–1.79, *p* < 0.001) over 3 years. The results were independent of anaemia status as there was no interaction between iron deficiency and anaemia. As such, the findings suggest that iron deficiency may be involved in low-grade inflammation even in relatively healthy older adults [116].

Consequently, chronic inflammatory conditions such as cancer, chronic infections, immune-mediated diseases, and obesity can also reduce RBCs. An estimated 40% of all anaemia cases worldwide are due to chronic disease or inflammation as a contributing cause [117]. Anaemia of inflammation (AI) or anaemia of chronic disease is the second most common type of anaemia after iron deficiency. Unlike absolute IDA, whereby the body’s iron store is depleted, the iron store of a patient with AI can remain normal. Hence, AI is also sometimes referred to as functional iron deficiency, which is characterised by the body’s inability to mobilise the available iron for erythropoiesis [118]. However, IDA and AI may co-exist in some patients, with anaemia due to inflammatory bowel disease being one example. As such, the diagnosis and management of IDA require a systematic evaluation of the case history and haematological profile, plus an investigation of the potential underlying causes of blood loss [115,119].

### 3.3. Treatment of IDA

Repleting iron stores is the primary strategy in IDA treatment, with prompt treatment needed to alleviate fatigue, improve quality of life, and reduce cognitive impairment [114]. Research evidence has shown that iron supplements are more effective than dietary iron for restoring iron status and Hb recovery [120,121]. A meta-regression analysis with data from 41 RCTs by Casgrain et al. [120] showed that iron supplementation affected serum ferritin (SF) concentration linearly related to duration (+0.51 µg/L per week, 95% CI: 0.02–1.00, *p* = 0.04) and dose (+0.10 µg/L per g Fe, 95% CI: 0.01–0.20, p=0.036). Hb concentration is also expected to increase significantly by 0.8 g/L for every 10 µg/L increase in baseline SF level with iron supplementation (p=0.02). It should be noted that these effects were observed in the healthy adult population as per the inclusion criteria of Casgrain et al. [120]. Another systematic review and meta-analysis by Houston et al. [122] further demonstrated that iron supplementation is more effective than placebo in reducing self-reported fatigue (standardised mean difference: −0.38, 95% CI: −0.52 to −0.23) among iron-deficient adults with no anaemia. Hence, for patients with iron deficiency or straightforward and uncomplicated IDA, oral iron supplement (ferrous sulphate, ferrous gluconate, or ferrous fumarate) is considered the standard care [123]. However, for IDA with complications such as intolerance, intestinal malabsorption, and ongoing blood losses that exceed iron absorption capacity, intravenous iron therapy may be needed to rapidly restore iron supply [124].

### 3.4. Adverse Effects of Oral Iron Therapy

While oral iron therapy as the first-line treatment for IDA is indisputable, considerable side effects are reported. A systematic review and meta-analysis of 43 RCTs by Tolkien et al. [9] confirmed that ferrous sulphate was 2.32 times (95% CI: 1.74–3.08, p<0.0001) and 3.05 times (95% CI: 2.07–4.48, p<0.0001) more likely to cause gastrointestinal side effects than placebo and intravenous iron therapy, respectively. Notably, meta-regression from this study did not find a significant relationship between the odds ratios of gastrointestinal side effects and dose. The common gastrointestinal side effects reported in the RCTs are constipation, nausea, diarrhoea, abdominal pain, vomiting, heartburn, dark stools, and flatulence [9].

There are also other negative impacts of oral iron therapy on the gastrointestinal tract besides symptoms of discomfort. Free iron, Fe^2^, is a divalent metal cation that can react with hydrogen peroxide in cells to produce hydroxyl radicals, which induce oxidative stress responses in cells. These radicals are cytotoxic to endothelial and smooth muscle cells and have deleterious impacts on health [110,125,126]. Hence, excess iron from oral iron supplements can lead to lipid peroxidation, which causes ferroptosis, mitochondrial damage, and endoplasmic reticulum dysfunction, leading to the destruction of the intestinal epithelial cells, affecting the intestinal mechanical barrier’s integrity [126]. Such are the possible underlying mechanisms of frequent gastrointestinal side effects of oral iron therapy. Moreover, anaemic patients taking oral iron supplements also demonstrated increased systemic oxidative stress as measured by lipid peroxide, protein carbonyl, conjugated dienes, lipid hydroperoxide and oxidised glutathione levels accompanied by a reduced total antioxidant level [127]. Hence, it is advisable to increase dietary antioxidant intake while taking oral iron supplements.

Excessive unabsorbed iron may cause gut dysbiosis, as it can modify gut microbiota composition, promoting the growth of pathogenic bacteria at the expense of the healthy ones [128,129,130]. Specifically, increased luminal iron concentration appeared to favour the growth of *Escherichia coli*, *Salmonella*, and *Bacteroides* species of pathogenic bacteria while lowering the abundance of probiotics such as *Lactobacillus* and *Bifidobacterium* species [128]. The disruption of gut microbiota equilibrium can also lead to inflammation and chronic conditions such as inflammatory bowel disease and metabolic dysfunction [128,131], which, in turn, can also hamper iron absorption and reduce the effectiveness of iron supplementation [132,133].

Overuse of iron supplements also carries considerable risks. Intake of iron above 60 mg/kg body weight can result in severe toxicity leading to gastrointestinal tract injury and impaired cellular metabolism in the heart, liver, and central nervous system, which can be fatal without immediate medical attention [134,135]. Moreover, prolonged ingestion of iron supplements for years has been reported to cause iron overload, where the body stores too much iron, a condition that can cause severe organ damage [136].

## 4. Ginger and IDA

The following sections will explore available evidence from in vivo, ex vivo, in vitro, and human clinical studies to elucidate the potential benefits of ginger for IDA and its associated clinical manifestations of altered iron metabolism. A summary of the research findings is presented in Table 2.

### 4.1. Iron Absorption Enhancement

Prakash and Srinivasan [137] demonstrated in an animal study that various spices (ginger, capsaicin, and piperine) can improve the bioavailability of dietary iron. Groups of Wistar rats were fed diets containing different spices for eight weeks before being sacrificed. Everted segments of duodenum, jejunum, and ileum of small intestines isolated from these rats were examined for ex vivo iron uptake through incubation with a medium containing finger millet powder fortified with iron. Compared to the control group that was not fed with any spices, all spice-fed groups showed significantly higher iron uptake percentages (p<0.05) in all sections of the small intestine. Ginger was the most potent among the three spices to increase iron uptake across all sections. The highest was 28.5 ± 2.09% uptake at the jejunum for ginger compared to 26.3 ± 1.30% for piperine, 22.6 ± 1.40% for capsaicin, and only 18.3 ± 0.39% for the control group. The suggested mechanism could be that the pungent spices altered mucosal permeation characteristics by increasing the absorptive surface [137].

Ginger can also be used as a food additive to enhance the bioavailability of non-haem iron. A study by Jaiswal et al. [138] compared the effects of iron bioaccessibility by adding various spices (ajwain, cumin, cinnamon, fennel, black pepper, and ginger) at 1 or 2% weight to wheat flour and different Indian bread formulations. The bioaccessibility of iron was measured through an in vitro dialysis method. Adding spices at 2% significantly enhanced iron bioaccessibility (*p* < 0.0001). Ginger, in particular, was shown to increase iron bioaccessibility in all food formulations by 2- to 3-folds. The authors attributed effects to the ascorbic acid and amino acids within ginger that favour iron absorption [138]. However, with its low vitamin C content (Table 1), iron bioaccessibility is likely to be assisted by other bioactive compounds in ginger.

The effects of ginger in enhancing iron availability have been confirmed in a human intervention study. Kulkarni et al. [139,140] conducted a human clinical study to demonstrate the potential use of ginger supplements in the treatment of IDA along with oral iron therapy. The study recruited 62 patients with anaemia following the WHO Hb cut-off levels, consisting of 12 males and 50 females, from a hospital in India. Their conditions were likely due to nutritional deficiency since those with chronic conditions, pregnancy, and blood donors were excluded. Participants were divided into two groups. The intervention group (n = 30) took 1.5 g of ginger powder with oral iron therapy, whereas the control group (n = 32) received only oral iron therapy as routine care. Fasting blood samples were collected at baseline and after 30 days. Pre- and post-treatment comparisons of haematological and iron parameters found significant increases (p<0.05) in all parameters in both groups. However, the intervention group achieved a more remarkable improvement in percentage difference than the control group in Hb (+8.23% vs. +2.3%), iron status (+19.63% vs. +5.54%), total iron-binding capacity (−7.23% vs. −4.47%), and SF (+45.11% vs. +34.11%). However, the authors did not report whether there were significant differences in the mean differences between groups. Notwithstanding, based on the published sample size, means, and standard deviations, the p-values could be easily estimated. The mean difference between groups for serum iron levels was significantly different post-treatment (p<0.007). Hence, the study demonstrated that ginger could assist in iron absorption and improve the efficacy of oral iron therapy for IDA.

The study by Kulkarni et al. [139,140] had several drawbacks. The first is its short duration of trial with only 30 days. Although patients with uncomplicated anaemia are expected to show improvement after 4 weeks of oral iron treatment, replenishment of iron store will take longer to achieve [123,124]. Secondly, the study did not track the adverse events experienced by the participants. Thus, there was no data on ginger’s effects on any side effects of oral iron therapy. Thirdly, there could be selection bias in the study design as it is unclear how the group assignment was carried out. Hence, the results from this study need further validation with more well-designed clinical trials.

### 4.2. Antioxidant Activity

Another study by Prakash and Srinivasan [141] showed that ginger can significantly enhance the activities of antioxidant enzymes (p<0.05), including superoxide dismutase (SOD), catalase (CAT), glutathione reductase (GR), and glutathione-S-transferase (GST), in both gastric and intestinal mucosa in vivo. For eight weeks, eight male Wistar rats were fed ad libitum with a basal diet enriched with 0.05% ginger powder. Compared to the control group provided with the basal diet only, the ginger group showed 48%, 11%, 67%, and 50% stimulation in the activities of CAT, SOD, GST, and GR, respectively, in the intestinal mucosa. In rats subjected to ethanol-induced oxidative stress, ginger treatment demonstrated higher SOD, GST, and GR activities in the gastric mucosa by 35%, 39%, and 30%, respectively, compared to controls. Moreover, the ginger-fed group also had 56% higher mucin content of gastric mucosa than the ethanol-treated controls. In short, this study illustrated the gastrointestinal protective effects of dietary ginger against oxidative stress.

The gingerol-related polyphenols and diarylheptanoids derived from the rhizomes of ginger possess remarkable free radical scavenging activities [142]. However, the antioxidant potency may vary across ginger varieties. In an in vitro experiment, Oboh et al. [143] studied the antioxidant effects of two types of gingers (red and white) against free iron radicals in rat brains. Although both variants possessed antioxidant capacities against Fe^2^, the study found red ginger (*Z. officinale* var. Rubra) superior to white ginger (*Z. officinale* Roscoe) at inhibiting Fe^2^-induced lipid peroxidation and chelating Fe^2^, likely due to its higher ascorbic acid, phenol, and flavonoid contents.

Hinneburg et al. [144] demonstrated in another study that the contents of total phenols in various spices had significant positive correlations with their antioxidant properties in terms of iron reduction (r^2^ = 0.8871, p<0.001) and inhibition of lipid peroxidation (r^2^ = 0.7327, p<0.01). Specifically, the hydro-distilled ginger extract showed relatively low Fe^3^ to Fe^2^ reducing activity and Fe^2^ chelating capacity compared to basil, parsley, juniper, cumin, and fennel extracts. The reduced antioxidant activities were attributed to the low total phenols/extractable compounds ratio of only 7.8% for ginger versus 59.7% for basil. The water-based extraction method used in Hinneburg et al. [144] could not preserve the antioxidant activity of essential oils from ginger. Hence, the variety, extraction and processing methods can greatly affect the antioxidant properties of ginger.

### 4.3. Anti-Inflammatory Action

The anti-inflammatory action of ginger and its potential application in AI were aptly reviewed by Kumar et al. [145]. Inflammation stimulates hepcidin production in response to pro-inflammatory cytokines such as IL-1, tumour necrotic factor (TNF)-α and, in particular, IL-6. As an acute phase protein, hepcidin’s role is to inhibit iron absorption and thus minimise the free iron supply to invading pathogens. Hence, sustained elevation of hepcidin will cause hypoferremia leading to anaemia. Additionally, an increase in IL-6 activates the nuclear factor kappa B (NF-κβ) pathway, resulting in the synthesis of CRP from the hepatocytes. The rising CRP levels indicate systemic inflammation and can blunt the erythropoiesis stimulation response in AI, especially in chronic kidney disease [145]. The bioactive compounds in ginger, such as 6-gingerol, 6-shogaol and 6-paradol, are known to possess broad anti-inflammatory properties that can block the activation of NF-κβ by suppressing pro-inflammatory cytokines [145,158].

The human intervention study on ginger and iron absorption reported by Kulkarni et al. [139,140] mentioned earlier also measured malondialdehyde (MDA) as the serum biomarker for oxidative stress and TNF-α as the inflammatory marker of the 62 participants receiving either ginger and iron treatment or oral iron therapy only. Both groups had an insignificant difference in mean MDA and TNF-α levels at baseline. After 30 days, both oxidative stress (MDA: −18.62%, p<0.001) and inflammatory markers (TNF-α: −20.11%, p<0.05) were significantly reduced in the oral iron plus ginger group. Conversely, in the control group taking only oral iron therapy, there was a significant decrease in post-trial MDA levels (−9.67%, p<0.05) and a non-significant increase in TNF-α (+3.86%, p>0.05) [140]. The estimated mean difference between groups was not significant in changes in MDA but was significant in TNF-α (p<0.05). It can be inferred from these results that, compared to oral iron therapy alone, combining ginger with oral iron therapy can better alleviate oxidative stress and reduce inflammation in patients with IDA while correcting their anaemic condition.

### 4.4. Gut Microbiota Modulation

Both iron deficiency and excess iron can lead to dysbiosis, characterised by an imbalance in the gut microbial community, and are associated with diseases [128]. Recent research has found ginger to exact prebiotic effects that improve gut microbiota composition. In a study that stimulated digestion and fermentation in vitro, 85% of the polyphenols in a dry ginger powder were still detectable in the digestive fluids after simulated digestion [146]. These polyphenol constituents include 6-, 8-, 10-gingerols and 6-shogaol. The undigested ginger extract significantly modulated faecal microbiota structure following mixed-culture fermentation with faecal inoculation compared with the control group. After 12 h of fermentation, the abundances of the beneficial bacterial groups of *Bifidobacterium* (p<0.05) and *Enterococcus* (p<0.01) were significantly higher in the ginger group than in the control group. The study also found elevated levels of short-chain fatty acids (SCFA) accompanied by decreased pH value after fermentation with ginger extract compared to control. The results demonstrated that ginger and its polyphenol compounds could improve human health through gut microbiota modulation [146].

Ginger’s effects on gut microbiota modulation were also confirmed in a mice model in an in vivo study [147]. Five-week-old C57BL/6J male mice were fed a high-fat diet with or without ginger supplementation for 16 weeks. With ginger treatment, mice on a high-fat diet showed lower body weight and amelioration of liver steatosis, low-grade inflammation, and insulin resistance compared to controls. Analysis of the gut microbiome showed an increase in *Bifidobacterium* genus and SCFA-producing bacteria (*Alloprevotella* and *Allobaculum*) and increases in faecal SCFA concentrations. As a high-fat diet promotes oxidative stress and chronic low-grade inflammation associated with metabolic dysfunction, this study demonstrated that ginger supplementation could mitigate the detrimental impact of a high-fat diet on gut microbiota composition to promote health.

Another in vivo study also found ginger to have therapeutic effects in relieving diarrhoea after antibiotic use through gut microbiota recovery [148]. The study used 5-week-old Sprague-Dawley rats treated with antibiotics by gavage for seven days before administering ginger extract for another seven days. The study found ginger treatment significantly reduced diarrhoea symptoms (p<0.05) compared to the control group that did not receive ginger treatment. Furthermore, the ginger treatment also considerably increased microbiota diversity in the gut, showing accelerated recovery. Specifically, the abundance of Proteobacteria phyla was increased by antibiotics treatment but restored significantly after ginger administration (p<0.0001). In contrast, antibiotic treatment depressed the abundance of Bacteroidetes phyla and saw significant improvement with ginger treatment (p<0.001). The study found *Escherichia Shigella* decreased at the genus levels, whereas Bacteroides increased the most in relative abundance. Histopathological observation of the colon also revealed evidence of intestinal barrier integrity improvement with ginger treatment associated with restoring tight junction protein Zonula occludens-1.

Changes in gut microbiota compositions in humans were also observed after consumption of ginger in a recent RCT [149]. The study recruited 138 healthy adults. All participants were advised to consume their usual diet but avoid ginger-rich products, probiotics, and prebiotics for one week before starting the study. During the intervention period, the participants were randomly assigned to take either fresh ginger juice (ginger group, n = 68) or sterile 0.9% sodium chloride (control group, n = 66) daily for seven days. Blood serum and faecal samples were collected at baseline and after seven days. A total of 4 participants in the control group and 7 in the ginger group were lost to follow-up, with only 123 participants completing the study. The study found increased counts of intestinal bacterial species when comparing the taxonomic composition between the ginger and control groups. The ginger juice intervention decreased the relative abundance of the Prevotella-to-Bacteroides ratio and the pro-inflammatory *Ruminococcus_1* and *Ruminococcus_2* genus and increased the Firmicutes-to-Bacteroidetes ratio, *Proteobacteria* and anti-inflammatory *Faecalibacterium*. Hence, the study concluded that consuming ginger juice for a short period had substantial effects on the composition and function of gut microbiota in healthy people.

There is currently no known pre-clinical and clinical study on the effect of ginger on the gut microbiota in the case of anaemia or iron deficiency. Nevertheless, prebiotics, such as inulin, are known to affect gut microbiota to improve iron absorption in IDA [130]. Thus, it is logical to assume a similar positive impact of ginger on IDA through inferences. This is an area of further research.

### 4.5. Erythropoiesis Stimulation

Erythropoiesis, the process of producing RBCs, is impeded in IDA due to insufficient dietary iron intake, impairment in iron absorption as affected by inflammation, or an imbalance between surging iron requirements and iron available resulting from rapid growth or heavy blood loss [159]. In fact, the use of ginger for haematopoiesis has an ethnomedicinal origin. Dry ginger is used in traditional Chinese medicine as a warm herb to promote blood flow, remove blood stasis, and alleviate weakness and fatigue. As such, ginger is used in many blood tonic herbal formulas for treating blood circulation, anaemia, and haemorheological conditions [160,161]. Ginger is also a tonic food recommended for postpartum women during the one month confinement period immediately after delivery for recovery from blood loss. Specifically, in Southern China, traditional postpartum dietary practices include the ‘ginger vinegar soup’ made from sweet vinegar, ginger, egg, and pig’s trotters [162]. This is an example of iron-rich food leveraging ginger as a functional ingredient to improve iron bioavailability and promote erythropoiesis. Thus, the ability of ginger to stimulate erythropoiesis can be an added benefit to the effectiveness of oral iron therapy for IDA treatment.

In a study that utilised zebrafish embryos to investigate the effect of ginger extract on haematopoiesis in vivo, Ferri-Lagneau et al. [150] found ginger, with its bioactive compounds of 8-gingerol, 10-gingerol, 8-shogaol, and 10-shogaol, promoted the expression of GATA-binding factor 1 (Gata1) in erythroid cells. Gata1 is an early marker and key regulator of erythropoiesis. Moreover, increases in the expression of haematopoietic progenitor markers cmyb and scl were also observed. The study also identified 10-gingerol as the most potent stimulator in promoting the primitive wave of erythropoiesis in early developing zebrafish embryos. The study further confirmed that the haematopoiesis effect of ginger was mediated through the bone morphogenetic protein (Bmp) signalling pathway.

In a subsequent study, the same group of researchers further demonstrated that ginger/10-gingerol can rescue the expression of haematopoietic stem/progenitor cells (HSPC) in zebrafish embryos with genetic defects [151]. Ginger was found to induce scl/runx1 expression through Bmp and Notch signalling pathways that led to arteriogenesis and HSPC formation. Bmp and Notch are known to regulate nitric oxide (NO) production, which plays an active role in the modulation of haematopoietic cell growth and differentiation. The study also showed that ginger produced a robust up-regulation of NO in the rescued mutant zebrafish embryos. Therefore, the combined effect of ginger on Bmp, Notch and NO production can be beneficial for the regulation of erythropoiesis for regeneration/recovery.

### 4.6. Iron Overload Prevention

Ginger’s ability in modulating iron absorption in the case of overload was an unexpected finding in a study that used ginger nanoparticle-derived lipid vectors (GDLV) to deliver DMT-1 short-interference RNAs (siRNA) that suppressed DMT-1 mRNA expression to reduce iron absorption in an iron-loading mice model [152]. The study found that GDLV containing negative control appeared to repress some iron-related parameters similar to the DMT-1 siRNA treatment. The observed effects were reductions of 20% in ^59^Fe absorption, approximately 65% of pancreatic non-haem iron, and 40 to 50% lower SF compared to controls. Hence, the authors suggested that the bioactive lipids in ginger could influence iron absorption and homeostasis.

In another animal model, Gholampour et al. [153] showcased the protective properties of ginger against the deleterious effects of iron overloading. To induce iron overload, male Wistar rats were given ferrous sulphate at 30 mg/kg/day, dissolved in 1 mL distilled water, intraperitoneally for 14 days. These rats showed significantly higher serum hepatic markers and bilirubin levels, elevated MDA levels, lower serum albumin levels, total protein, triglyceride, cholesterol, and glucose, decreased creatinine clearance and higher fractional excretion of sodium compared to controls (p<0.001). The histopathological examination further confirmed their liver and kidney damage. A separate group of iron overloaded rats was fed with a hydroalcoholic ginger extract at 400 mg/kg/day dissolved in 1 mL distilled water and given by gavage for 11 days from the fourth day of ferrous sulphate injection. The feeding of ginger markedly reversed the adverse impacts of iron overload, as evidenced in the significantly higher levels of hepatic serum markers, renal functional markers and lipid peroxidation markers in this group compared to the iron only group (p<0.01). Moreover, depleted serum total protein, albumin, glucose, triglycerides, and cholesterol were restored with bilirubin concentration decreased in the blood. Hence, ginger extract demonstrated strong protective effects against iron toxicity potentially through its free radical scavenging activities. The preservation of the liver and kidney was also corroborated through histological examinations.

The potential of ginger, especially its 6-shogaol derivative, in preventing iron overload is further demonstrated in a case series reported by Golombick et al. [154]. In this study, 6 early-stage, transfusion-independent patients with myelodysplastic syndrome (MDS) were given a daily supplement of 20 mg of a ginger extract standardised for 20% 6-shogaol. Blood and urine samples were collected monthly. At three months, the study found that 6-shogaol was able to reduce the SF levels (>40% reductions) of three of the patients who had elevated SF (>300 g/µL) at baseline. Two of the patients who had SF reduction repeated the study for another 3 months after a washout period. Again, a greater than 40% reduction in SF was observed in the repeat tests for both patients. The two patients were tested for their serum hepcidin levels at the repeat tests. Both patients demonstrated elevation of serum hepcidin that accompanied the SF reduction. Furthermore, one patient who had high liver function enzymes due to alcohol consumption also saw normalisation of liver function with a greater than 40% reduction in these enzymes at the end of the study. The restoration of liver function was achieved without changing alcohol consumption habits. The research concluded that ginger extract rich in 6-shogaol prevented iron overload in MDS patients through upregulation of hepcidin, potentially with liver function restoration.

### 4.7. Ginger-Synthesised Iron Nanoparticles

Nanotechnology, the understanding and control of matter generally in the 1–100 nm dimension, is gaining much medical research as it holds the potential for breakthroughs in preventing, diagnosing, and treating various diseases due to the unique physicochemical properties of nanomaterials [163,164]. Unsurprisingly, iron nanoparticle (FeNP) preparations have also been developed to overcome the inherent limitations of conventional ferrous and ferric iron formulations in the treatment of IDA. Pre-clinical studies showed that iron nanoparticles have high bioavailability, are non-toxic, and induce lesser side effects than conventional iron preparations for IDA, even though the delivery and safety issues in humans for therapeutic use required further research [165]. The high bioavailability of iron nanoparticles is also ideal for food fortifications as the nanoparticles do not cause unacceptable taste or colour in food vehicles. Hence, it is suggested that nanosized iron salts can have potential applications in food fortification to reduce IDA worldwide [166].

Ginger has been used in the green approach for metallic nanoparticles, including iron. The green synthesis approach is preferred to avoid the production of unwanted or harmful chemical by-products and achieve a cost-effective and sustainable supply of nanoparticles [167]. El-Refai et al. [155] used ginger and garlic extracts to synthesise silver, copper, iron, and zinc nanoparticles, and their antioxidant and antimicrobial activities were evaluated. The high flavonoid and phenolic contents in garlic and ginger water extracts revealed in the phytochemical analysis strongly support the potential of garlic and ginger to bio-reduce the metallic ions to their respective nanoparticles (e.g., Fe^3+^ ions to FeNPs). Transmission electron microscopy showed that the FeNPs in ginger were in the range of 14.08–21.57 nm with almost spherical forms. In comparison, the particle size of FeNPs in garlic ranged from 60.30 to 82.63 nm with tetragonal structures. All nanoparticles extracted in this study, including FeNPs from ginger, demonstrated considerable radical scavenging properties and antimicrobial activities against Gram-positive and Gram-negative bacteria and fungi [155]. Other researchers have similarly shown ginger to be a suitable green material for synthesising FeNPs with high antioxidant and antibacterial properties [156,157].

## 5. Conclusions

In summary, ginger with its rich polyphenols can support IDA treatment and prevention in many ways. It can improve iron bioavailability by enhancing iron absorption and thus increasing the efficacy of oral iron therapy. Its antioxidant and anti-inflammatory properties help in reducing oxidative stress and pro-inflammatory cytokine cascade and thus protect the gastrointestinal tract from the delirious effects of excess free iron. Ginger and its bioactive polyphenols can also serve as prebiotics to the gut microbiota to promote gut health and potentially reduce the unwanted side effects of oral iron therapy. Ginger can also stimulate erythropoiesis to generate the much-needed healthy RBCs for proper functioning. In the case of iron overload due to comorbidities from inflammatory disorders or chronic conditions, ginger can potentially reverse the adverse impacts and restore iron balance. Ginger can also be used to synthesise FeNPs sustainably to develop newer and more effective oral iron products and functional ingredients for IDA treatment and prevention.

There are, however, still many unknowns regarding the physiological effects of ginger and its active compounds in IDA. Much research is still needed to understand how the phenolic compounds of ginger can influence the mechanistic pathways of iron absorption and metabolism. More pre-clinical studies are required to further explore how ginger’s antioxidant, anti-inflammatory, gut microbiota modulation, and erythropoiesis stimulation properties can affect IDA in areas such as side effects induced by oral iron therapy, gastrointestinal micro-environment and microbiota changes, inflammatory cytokine signalling and erythropoiesis effectiveness. Most importantly, there is a lack of clinical study on the effect of co-administration of ginger and oral iron therapy for IDA treatment other than one short human trial. There is a need for a longer-term, randomised, double-blind placebo-controlled trial to validate the effectiveness of ginger as an adjuvant treatment for IDA.

To conclude, polyphenol-rich ginger can play a much bigger role in addressing the global public health problem of IDA, but more research and development are needed to realise its full potential.

## Figures and Tables

**Table 1:** Nutritional composition of dried ginger powder as reported by Ajayi et al. [19] and Sangwan et al. [20].

Nutrient	Amount	Unit
Carbohydrate	39.70–58.21	%
Protein	11.65–12.05	%
Crude fibre	8.30–21.90	%
Fat	9.89–17.11	%
Moisture	3.95–4.63	%
Ash	4.95–7.45	%
β-carotene	0.68–0.81	mg/100 g
Ascorbic acid	2.2–3.8	mg/100 g
Polyphenols	11.8–12.5	mg/100 g
Calcium	64.4–69.2	mg/100 g
Iron	1.5–1.8	mg/100 g
Copper	0.46–0.75	mg/100 g

**Table 2:** A summary of the research findings on ginger’s beneficial properties in its applications for iron deficiency anaemia and associated clinical manifestations of altered iron metabolism.

Beneficial Property	Study Type	Research Findings	Reference
Iron absorption enhancement	Ex vivo	Ginger was the most potent spice for enhancing iron absorption by increasing uptake by 28.5 ± 2.09% in the jejunum of rats compared to control.	[137]
	In vitro	Adding ginger to food enhanced the bioaccessibility of dietary iron by 2- to 3-fold depending on the formulations.	[138]
	Human study	Ginger plus oral iron therapy improved haematological and iron parameters of anaemic patients better than oral iron therapy alone.	[139,140]
Antioxidant activity	In vivo	Adding ginger to the diet significantly increased the activities of antioxidant enzymes (p<0.05) at the intestinal and gastric mucosa of rats, demonstrating enhanced protective effects against oxidative stress.	[141]
	In vitro	The polyphenols and diarylheptanoid derivatives of ginger contributed to both radical scavenging and inhibitory effects of autoxidation.	[142]
	In vitro	Both red and white ginger variants possessed antioxidant capacities against free iron radicals in rat brains, but red ginger was superior at inhibiting Fe^2^-induced lipid peroxidation and chelating Fe^2^.	[143]
	In vitro	Water-based extract of ginger showed relatively low antioxidant activities compared to other spices due to reduced phenolic contents produced from hydro-distillation extraction.	[144]
Anti-inflammatory action	Review	The bioactive compounds in ginger possessed broad anti-inflammatory properties that can block the activation of NF-κβ by suppressing pro-inflammatory cytokines of IL-1, TNF-α and IL-6, thus preventing hepcidin production.	[145]
	Human study	Ginger plus oral iron therapy significantly reduced the inflammatory marker TNF-α (p<0.05) in anaemic patients better than oral iron therapy alone.	[139,140]
Gut microbiota modulation	In vitro	Undigested ginger polyphenols significantly increased the abundances of *Bifidobacterium* (p<0.05) and *Enterococcus* (p<0.01) after faecal inoculated fermentation, accompanied by elevated levels of SCFA and decreased pH value.	[146]
	In vivo	Ginger supplementation could mitigate the detrimental impact of a high-fat diet in mice by promoting the abundance of *Bifidobacterium* genus and SCFA-producing bacteria (*Alloprevotella* and *Allobaculum*).	[147]
	In vivo	Ginger treatment significantly reduced antibiotic-associated diarrhoea symptoms (p<0.05) in rats with an associated increase in microbiota diversity and improved intestinal barrier integrity.	[148]
	Human study	Ginger juice consumption in healthy adults decreased the Prevotella-to-Bacteroides ratio and pro-inflammatory *Ruminococcus_1 and* *Ruminococcus_2* genus while increasing the Firmicutes-to-Bacteroidetes ratio, Proteobacteria and anti-inflammatory *Faecalibacterium*.	[149]
Erythropoiesis stimulation	In vivo	Ginger, with its bioactive compounds of 8-gingerol, 10-gingerol, 8-shogaol, and 10-shogaol, promoted the expression of Gata1 in erythroid cells of zebrafish embryos through the Bmp signalling pathway.	[150]
	In vivo	Ginger induced scl/runx1 expression through Bmp and Notch signalling pathways which up-regulated nitric oxide production for regeneration of haematopoietic stem/progenitor cells.	[151]
Iron overload prevention	In vivo	The bioactive lipids in ginger repressed some iron-related parameters, including reductions in 20% of ^59^Fe absorption, 65% of pancreatic non-haem iron, and 40% to 50% of serum ferritin levels, compared to controls.	[152]
	In vivo	Ginger extract demonstrated strong protective effects against iron toxicity through its free radical scavenging activities in iron-overloaded rats.	[153]
	Case series	Ginger extract rich in 6-shogaol prevented iron overload in three patients with myelodysplastic syndrome. These patients had elevated serum ferritin (>300 g/μL) at baseline but achieved >40% reductions after three months through upregulation of hepcidin.	[154]
Ginger-synthesised iron nanoparticles	In vitro	Ginger was used to bio-reduce the metallic ions to nanoparticles (Fe^3+^ ions to FeNPs). Transmission electron microscopy showed that the FeNPs in ginger were in the range of 14.08–21.57 nm with almost spherical forms and demonstrated considerable radical scavenging properties and antimicrobial activities against Gram-positive and Gram-negative bacteria and fungi.	[155]
	In vitro	Ginger can be a suitable green material for synthesising iron nanoparticles with high antioxidant and antibacterial properties.	[156,157]

Abbreviations: bone morphogenetic protein (Bmp); GATA-binding factor 1 (Gata1); interleukin (IL); short-chain fatty acids (SCFA); tumour necrosis factor alpha (TNF-*α*).

## Data Availability

Not applicable.

## Abbreviations

The following abbreviations are used in this manuscript:

AI	Anaemia of inflammation
CAT	Catalase
CI	Confidence interval
CRP	C-reactive protein
DcytB	Duodenal cytochrome B
DMT-1	Divalent metal transporter 1
FeNP	Iron nanoparticle
FPN	Ferroportin
Gata1	GATA-binding factor 1
GR	Glutathione reductase
GST	Glutathione-S-transferase
HAMP	Hepcidin antimicrobial peptide
Hb	Haemoglobin
HSPC	Haematopoietic stem/progenitor cells
IDA	Iron deficiency anaemia
MDA	Malondialdehyde
NFκβ	Nuclear factor kappa B
NO	Nitric oxide
NOAEL	No observed adverse effect level
RBC	Red blood cell
RCT	Randomised controlled trial
SF	Serum ferritin
siRNA	Short interference RNA
SOD	Superoxide dismutase
TNF	Tumour necrotic factor
WHO	World Health Organization

## Appendix A. International Patents

The following table summarises all patent publications (total = 16) found using the World Intellectual Property Organization online PATENTSCOPE database with the search term (Ginger AND “Iron deficiency”).

**Table A1:** A summary list of patent publications related to functional foods or ethnomedicine indicated for iron deficiency with ginger as an ingredient.

Publication No	Date	Classification Code	Title	Country
101243891	20 August 2008	A23L 1/337	Sea tangle vegetarian stuffing boiled dumplings and its processing method	China
103947928	11 March 2014	A23L 1/10	Fleece-flower root nutrition eight-treasure porridge and its preparation method	China
104026495	10 September 2014	A23L 1/212	Haw flake containing pig blood and coarse cereals, and preparation method thereof	China
105495158	25 September 2014	A23L 1/315	Black-bone chicken sausage and preparation method thereof	China
104095016	15 October 2014	A61K 36/9068	Infantile iron-deficiency anemia treating cookie and preparing method thereof	China
104323303	4 November 2015	A23L 1/314	Method for making tomato beef stewed product	China
104643216	27 May 2015	A23L 2/02	Blood-replenishing and beautifying calcium blended lotus root juice and preparation method thereof	China
105664116	15 June 2016	A61K 36/9068	Traditional Chinese medicine for treating infant iron deficiency anemia as well as preparation method and application thereof	China
106362108	1 February 2017	A61K 36/9068	Traditional Chinese medicinal pill used for hematogenesis	China
106616937	10 May 2017	A23L 31/00	Stropharia rugosoannulata and black chicken can and preparation method thereof	China
106889422	27 June 2017	A61K 36/9068	Edible flour for tonifying blood and warming the uterus and production method thereof	China
107772293	9 March 2018	A23L 13/50	Body-nourishing black bone chicken	China
107772373	9 March 2018	A61K 36/9068	Decoction preventing and curing osteoporosis	China
108887607	27 November 2018	A23L 13/70	Spicy shredded pork with garlic sauce and making method thereof	China
108433084	24 August 2018	A23L 27/50	Soy sauce	China
113278488	20 August 2021	A61K 36/9068	Spartina alterniflora spleen-tonifying stomach-nourishing pericarpium citri reticulatae wine decocting pot and preparation process thereof	China
